# Dichlorido{2-[(2,6-dimethyl­phen­yl)imino­meth­yl]pyridine-κ^2^ *N*,*N*′}zinc

**DOI:** 10.1107/S1600536812006204

**Published:** 2012-02-24

**Authors:** Xue-hong Liu, Li-min Zhao, Feng-shou Liu

**Affiliations:** a School of Chemistry and Chemical Engineering, GuangDong Pharmaceutical University, Guangzhou 510006, People’s Republic of China

## Abstract

In the asymmetric unit of the title compound, [ZnCl_2_(C_14_H_14_N_2_)], the central Zn^II^ ion is four-coordinated in a distorted tetra­hedral environment by two N atoms of the ligand 2-[(2,6-dimethyl­phen­yl)imino­meth­yl]pyridine and two chloride anions. In the crystal, adjacent mol­ecules are connected through C—H⋯Cl hydrogen bonds between a C—H group of the ligand and a Cl^−^ anion, leading to a chain-like structure along the *b* direction.

## Related literature

For related structures, see: Roy *et al.* (2011 ▶); Shi *et al.* (2010 ▶); Talei Bavil Olyai *et al.* (2008 ▶); Schulz *et al.* (2009 ▶); Hathwar *et al.*. (2010 ▶).
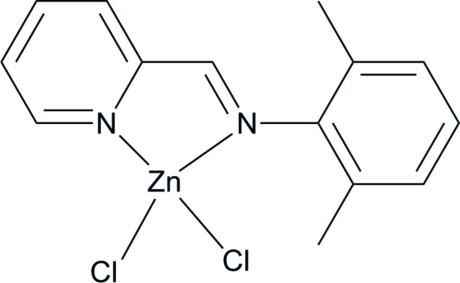


## Experimental

### 

#### Crystal data

[ZnCl_2_(C_14_H_14_N_2_)]
*M* *_r_* = 346.54
Monoclinic, 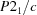

*a* = 14.360 (4) Å
*b* = 8.222 (2) Å
*c* = 13.176 (4) Å
β = 105.770 (3)°
*V* = 1497.0 (7) Å^3^
*Z* = 4
Mo *K*α radiation
μ = 1.98 mm^−1^
*T* = 296 K
0.80 × 0.60 × 0.60 mm

#### Data collection

Bruker APEXII CCD diffractometer
Absorption correction: multi-scan (*SADABS*; Bruker, 2001 ▶) *T*_min_ = 0.300, *T*_max_ = 0.382
7309 measured reflections
2620 independent reflections
2099 reflections with *I* > 2σ(*I*)
*R*_int_ = 0.027

#### Refinement

*R*[*F* ^2^ > 2σ(*F* ^2^)] = 0.030
*wR*(*F* ^2^) = 0.071
*S* = 1.01
2620 reflections
174 parameters
H-atom parameters constrained
Δρ_max_ = 0.34 e Å^−3^
Δρ_min_ = −0.34 e Å^−3^

### 

Data collection: *APEX2* (Bruker, 2001 ▶); cell refinement: *SAINT-Plus* (Bruker, 2001 ▶); data reduction: *SAINT-Plus*; program(s) used to solve structure: *SHELXS97* (Sheldrick, 2008 ▶); program(s) used to refine structure: *SHELXL97* (Sheldrick, 2008 ▶); molecular graphics: *SHELXTL* (Sheldrick, 2008 ▶); software used to prepare material for publication: *SHELXTL*.

## Supplementary Material

Crystal structure: contains datablock(s) I, global. DOI: 10.1107/S1600536812006204/zj2055sup1.cif

Structure factors: contains datablock(s) I. DOI: 10.1107/S1600536812006204/zj2055Isup2.hkl

Additional supplementary materials:  crystallographic information; 3D view; checkCIF report

## Figures and Tables

**Table 1:** Hydrogen-bond geometry (Å, °)

*D*—H⋯*A*	*D*—H	H⋯*A*	*D*⋯*A*	*D*—H⋯*A*
C4—H4⋯Cl1^i^	0.93	2.95	3.762 (3)	147
C6—H6⋯Cl1^i^	0.93	2.85	3.675 (3)	148
C1—H1⋯Cl2^ii^	0.93	2.93	3.684 (3)	139

Symmetry codes: (i) 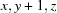; (ii) 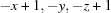.

**Fig. 1.**
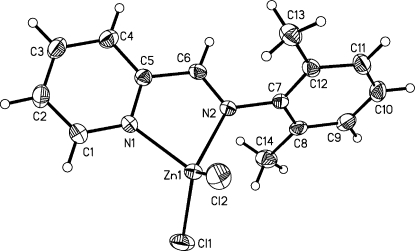
The molecular structure of the title compound drawn with 50% ellipsoidal probability.

**Fig. 2.**
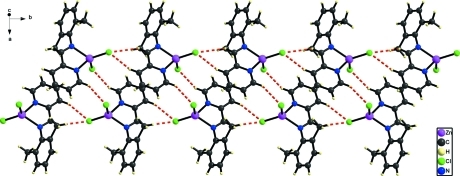
The one-dimensional chain-like structure connected through the C—H···Cl inter-molecule hydrogen bonds.

## supplementary crystallographic information

### Comment

Recently, the bidentate [N, N] ligand such as pyridineimine have drawn much attention owing to their valuable applications in the fields of catalysis, conjugated organic devices. These bidentate ligands can be modified by tuning the substituents. Therefore, different steric and electronic properties are achieved easily. Various zinc metal complexes (Roy *et al.* 2011; Shi *et al.* 2010; Talei Bavil Olyai *et al.* 2008; Schulz *et al.* 2009) have been developed. In order to enrich this family type of compounds, we report the single-crystal growth and structure investigation of title compound [Zn(C_14_H_14_N_2_)Cl_2_].

The molecular structure of the compound is shown in Fig. 1. The solid-state structure showed a distorted tetrahedral coordinate geometry formed by two N atoms from the ligand 2,6-dimethyl-*N*-(pyridine-2-ylmethylene)aniline and two chloride atoms, with the Zn—N distances of 2.071 (2) and 2.078 (2) Å and the Zn—Cl distances of 2.1972 (10) and 2.2135 (11) Å. On an over view (Fig. 2), the adjacent molecules were connected through the C—H···Cl inter-molecule hydrogen bonds between the C—H group of the ligand and the Cl atom, leading to a one-dimensional chain-like structure.

### Experimental

A mixture of picolinaldehyde (0.0535 g, 0.5 mmol) and 2,6-dimethylaniline (0.0606 g, 0.5 mmol) was refluxed in CH_3_OH (20 ml) for 2 h, ZnCl_2_ (0.0682 g, 0.5 mmol) was added and refluxed for another 30 min, then cooled to the room temperature gradually, yellow precipitates were obtained at this time, which were dissolved in the solution of DMSO (5 ml) and CH_3_OH (3 ml). After the evaporation process at room temperature for about 12 d, yellow crystals were got.

### Refinement

X-ray data were collected on a *APEX2* (Bruker, 2001).Semi-empirical absorption corrections were made using *SADABS*. The structures were solved using direct methods, followed by full matrix least-squares refinement against *F*^2^ (all data) using *SHELXTL*. Anisotropic refinement for all ordered non-H atoms; organic H atoms were placed in calculated positions.

### Crystal data

**Table:**

[ZnCl_2_(C_14_H_14_N_2_)]	*F*(000) = 704
*M**_r_* = 346.54	*D*_x_ = 1.538 Mg m^−^^3^
Monoclinic, *P*2_1_/*c*	Mo *K*α radiation, λ = 0.71073 Å
*a* = 14.360 (4) Å	Cell parameters from 2535 reflections
*b* = 8.222 (2) Å	θ = 2.9–25.3°
*c* = 13.176 (4) Å	µ = 1.98 mm^−^^1^
β = 105.770 (3)°	*T* = 296 K
*V* = 1497.0 (7) Å^3^	Block, yellow
*Z* = 4	0.80 × 0.60 × 0.60 mm

### Data collection

**Table:**

Bruker APEXII CCD diffractometer	2620 independent reflections
Radiation source: fine-focus sealed tube	2099 reflections with *I* > 2σ(*I*)
Graphite monochromator	*R*_int_ = 0.027
φ and ω scans	θ_max_ = 25.0°, θ_min_ = 2.9°
Absorption correction: multi-scan (*SADABS*; Bruker, 2001)	*h* = −16→17
*T*_min_ = 0.300, *T*_max_ = 0.382	*k* = −8→9
7309 measured reflections	*l* = −15→15

### Refinement

**Table:**

Refinement on *F*^2^	Primary atom site location: structure-invariant direct methods
Least-squares matrix: full	Secondary atom site location: difference Fourier map
*R*[*F*^2^ > 2σ(*F*^2^)] = 0.030	Hydrogen site location: inferred from neighbouring sites
*wR*(*F*^2^) = 0.071	H-atom parameters constrained
*S* = 1.01	*w* = 1/[σ^2^(*F*_o_^2^) + (0.0267*P*)^2^ + 0.7932*P*] where *P* = (*F*_o_^2^ + 2*F*_c_^2^)/3
2620 reflections	(Δ/σ)_max_ = 0.002
174 parameters	Δρ_max_ = 0.34 e Å^−^^3^
0 restraints	Δρ_min_ = −0.34 e Å^−^^3^

### Special details

**Table:**

Geometry. All e.s.d.'s (except the e.s.d. in the dihedral angle between two l.s. planes) are estimated using the full covariance matrix. The cell e.s.d.'s are taken into account individually in the estimation of e.s.d.'s in distances, angles and torsion angles; correlations between e.s.d.'s in cell parameters are only used when they are defined by crystal symmetry. An approximate (isotropic) treatment of cell e.s.d.'s is used for estimating e.s.d.'s involving l.s. planes.
Refinement. Refinement of *F*^2^ against ALL reflections. The weighted *R*-factor *wR* and goodness of fit *S* are based on *F*^2^, conventional *R*-factors *R* are based on *F*, with *F* set to zero for negative *F*^2^. The threshold expression of *F*^2^ > σ(*F*^2^) is used only for calculating *R*-factors(gt) *etc*. and is not relevant to the choice of reflections for refinement. *R*-factors based on *F*^2^ are statistically about twice as large as those based on *F*, and *R*-factors based on ALL data will be even larger.

### Fractional atomic coordinates and isotropic or equivalent isotropic displacement parameters (Å^2^)

**Table:**

	*x*	*y*	*z*	*U*_iso_*/*U*_eq_	
Zn1	0.30152 (2)	0.11718 (4)	0.46883 (3)	0.03949 (12)	
C1	0.50567 (19)	0.2130 (4)	0.6054 (2)	0.0479 (7)	
H1	0.5253	0.1072	0.5970	0.058*	
C2	0.5729 (2)	0.3225 (4)	0.6611 (3)	0.0543 (8)	
H2	0.6364	0.2901	0.6916	0.065*	
C3	0.5449 (2)	0.4803 (4)	0.6712 (2)	0.0526 (8)	
H3	0.5897	0.5568	0.7066	0.063*	
C4	0.4498 (2)	0.5235 (4)	0.6283 (2)	0.0458 (7)	
H4	0.4291	0.6294	0.6344	0.055*	
C5	0.38552 (19)	0.4066 (3)	0.5760 (2)	0.0354 (6)	
C6	0.28131 (19)	0.4390 (3)	0.5312 (2)	0.0365 (6)	
H6	0.2565	0.5420	0.5373	0.044*	
C7	0.12475 (18)	0.3613 (3)	0.4352 (2)	0.0347 (6)	
C8	0.05578 (19)	0.2822 (3)	0.4741 (2)	0.0384 (7)	
C9	−0.0408 (2)	0.3138 (4)	0.4244 (3)	0.0489 (8)	
H9	−0.0886	0.2641	0.4490	0.059*	
C10	−0.0669 (2)	0.4178 (4)	0.3393 (3)	0.0545 (9)	
H10	−0.1320	0.4373	0.3069	0.065*	
C11	0.0025 (2)	0.4922 (4)	0.3023 (2)	0.0490 (8)	
H11	−0.0163	0.5613	0.2445	0.059*	
C12	0.10028 (19)	0.4669 (3)	0.3492 (2)	0.0394 (7)	
C13	0.1757 (2)	0.5507 (4)	0.3071 (2)	0.0546 (8)	
H13A	0.2012	0.6425	0.3508	0.082*	
H13B	0.1466	0.5870	0.2363	0.082*	
H13C	0.2270	0.4759	0.3073	0.082*	
C14	0.0848 (2)	0.1719 (4)	0.5686 (3)	0.0541 (8)	
H14A	0.1171	0.0778	0.5514	0.081*	
H14B	0.0282	0.1386	0.5885	0.081*	
H14C	0.1278	0.2290	0.6261	0.081*	
Cl1	0.28638 (6)	−0.11449 (9)	0.54595 (8)	0.0664 (3)	
Cl2	0.31417 (6)	0.11215 (10)	0.30514 (6)	0.0610 (2)	
N1	0.41315 (14)	0.2537 (3)	0.56299 (17)	0.0372 (5)	
N2	0.22550 (14)	0.3265 (2)	0.48443 (16)	0.0319 (5)	

### Atomic displacement parameters (Å^2^)

**Table:**

	*U*^11^	*U*^22^	*U*^33^	*U*^12^	*U*^13^	*U*^23^
Zn1	0.03867 (19)	0.02821 (18)	0.0486 (2)	0.00313 (14)	0.00686 (15)	−0.00420 (15)
C1	0.0391 (16)	0.0441 (18)	0.058 (2)	0.0072 (14)	0.0085 (14)	0.0018 (15)
C2	0.0352 (16)	0.062 (2)	0.060 (2)	−0.0025 (15)	0.0026 (15)	0.0056 (17)
C3	0.0437 (18)	0.055 (2)	0.054 (2)	−0.0138 (15)	0.0047 (15)	−0.0060 (16)
C4	0.0460 (17)	0.0403 (17)	0.0492 (19)	−0.0061 (14)	0.0099 (14)	−0.0073 (14)
C5	0.0373 (14)	0.0338 (15)	0.0341 (15)	−0.0008 (12)	0.0080 (12)	0.0007 (12)
C6	0.0402 (15)	0.0283 (14)	0.0409 (16)	0.0049 (12)	0.0106 (13)	−0.0023 (12)
C7	0.0337 (14)	0.0268 (14)	0.0408 (16)	0.0025 (11)	0.0055 (12)	−0.0067 (12)
C8	0.0415 (16)	0.0264 (14)	0.0473 (17)	−0.0014 (12)	0.0121 (13)	−0.0112 (12)
C9	0.0374 (16)	0.0419 (17)	0.069 (2)	−0.0048 (14)	0.0172 (15)	−0.0166 (16)
C10	0.0353 (16)	0.053 (2)	0.066 (2)	0.0099 (15)	−0.0012 (15)	−0.0161 (17)
C11	0.0480 (18)	0.0470 (18)	0.0459 (19)	0.0127 (15)	0.0023 (15)	−0.0020 (14)
C12	0.0409 (15)	0.0358 (15)	0.0393 (17)	0.0070 (13)	0.0072 (13)	−0.0034 (13)
C13	0.0586 (19)	0.0528 (19)	0.052 (2)	0.0047 (16)	0.0135 (16)	0.0106 (16)
C14	0.0572 (19)	0.0446 (17)	0.066 (2)	−0.0003 (15)	0.0270 (17)	0.0063 (16)
Cl1	0.0729 (6)	0.0314 (4)	0.0953 (7)	0.0023 (4)	0.0236 (5)	0.0090 (4)
Cl2	0.0668 (5)	0.0669 (5)	0.0487 (5)	0.0093 (4)	0.0148 (4)	−0.0096 (4)
N1	0.0327 (12)	0.0325 (12)	0.0436 (14)	0.0044 (10)	0.0057 (10)	0.0014 (10)
N2	0.0330 (11)	0.0275 (11)	0.0346 (12)	0.0026 (10)	0.0082 (10)	−0.0005 (10)

### Geometric parameters (Å, º)

**Table:**

Zn1—N1	2.071 (2)	C7—C12	1.394 (4)
Zn1—N2	2.078 (2)	C7—N2	1.444 (3)
Zn1—Cl1	2.1972 (10)	C8—C9	1.387 (4)
Zn1—Cl2	2.2135 (11)	C8—C14	1.504 (4)
C1—N1	1.336 (3)	C9—C10	1.379 (4)
C1—C2	1.377 (4)	C9—H9	0.9300
C1—H1	0.9300	C10—C11	1.367 (4)
C2—C3	1.375 (4)	C10—H10	0.9300
C2—H2	0.9300	C11—C12	1.388 (4)
C3—C4	1.376 (4)	C11—H11	0.9300
C3—H3	0.9300	C12—C13	1.511 (4)
C4—C5	1.379 (4)	C13—H13A	0.9600
C4—H4	0.9300	C13—H13B	0.9600
C5—N1	1.343 (3)	C13—H13C	0.9600
C5—C6	1.476 (4)	C14—H14A	0.9600
C6—N2	1.269 (3)	C14—H14B	0.9600
C6—H6	0.9300	C14—H14C	0.9600
C7—C8	1.394 (4)		
			
N1—Zn1—N2	80.43 (8)	C10—C9—C8	121.1 (3)
N1—Zn1—Cl1	110.53 (7)	C10—C9—H9	119.5
N2—Zn1—Cl1	123.47 (7)	C8—C9—H9	119.5
N1—Zn1—Cl2	109.80 (7)	C11—C10—C9	120.2 (3)
N2—Zn1—Cl2	107.24 (6)	C11—C10—H10	119.9
Cl1—Zn1—Cl2	118.63 (4)	C9—C10—H10	119.9
N1—C1—C2	122.1 (3)	C10—C11—C12	121.4 (3)
N1—C1—H1	118.9	C10—C11—H11	119.3
C2—C1—H1	118.9	C12—C11—H11	119.3
C3—C2—C1	119.2 (3)	C11—C12—C7	117.1 (3)
C3—C2—H2	120.4	C11—C12—C13	120.5 (3)
C1—C2—H2	120.4	C7—C12—C13	122.4 (2)
C2—C3—C4	119.2 (3)	C12—C13—H13A	109.5
C2—C3—H3	120.4	C12—C13—H13B	109.5
C4—C3—H3	120.4	H13A—C13—H13B	109.5
C3—C4—C5	118.7 (3)	C12—C13—H13C	109.5
C3—C4—H4	120.7	H13A—C13—H13C	109.5
C5—C4—H4	120.7	H13B—C13—H13C	109.5
N1—C5—C4	122.3 (2)	C8—C14—H14A	109.5
N1—C5—C6	114.9 (2)	C8—C14—H14B	109.5
C4—C5—C6	122.8 (2)	H14A—C14—H14B	109.5
N2—C6—C5	120.0 (2)	C8—C14—H14C	109.5
N2—C6—H6	120.0	H14A—C14—H14C	109.5
C5—C6—H6	120.0	H14B—C14—H14C	109.5
C8—C7—C12	122.8 (2)	C1—N1—C5	118.4 (2)
C8—C7—N2	117.9 (2)	C1—N1—Zn1	129.37 (19)
C12—C7—N2	119.3 (2)	C5—N1—Zn1	112.12 (16)
C9—C8—C7	117.3 (3)	C6—N2—C7	119.8 (2)
C9—C8—C14	121.3 (3)	C6—N2—Zn1	111.88 (17)
C7—C8—C14	121.4 (2)	C7—N2—Zn1	127.51 (16)

### Hydrogen-bond geometry (Å, º)

**Table:**

*D*—H···*A*	*D*—H	H···*A*	*D*···*A*	*D*—H···*A*
C4—H4···Cl1^i^	0.93	2.95	3.762 (3)	147
C6—H6···Cl1^i^	0.93	2.85	3.675 (3)	148
C1—H1···Cl2^ii^	0.93	2.93	3.684 (3)	139

Symmetry codes: (i) *x*, *y*+1, *z*; (ii) −*x*+1, −*y*, −*z*+1.
